# Supplementary material for: Chinese health funding in Africa: The untold story
Source: PLOS Glob Public Health. 2023 Jun 28;3(6):e0001637. doi: 10.1371/journal.pgph.0001637 (PMC10306200; doi:10.1371/journal.pgph.0001637)
Supplement: Attachment — Submitted filename: R&R_PLOS_One.docx [file pgph.0001637.s001.docx]

Dear Editor and Reviewers,

Thank you for your feedback on this piece; it was immensely helpful in crafting this minor revision, and has helped us clarify a number of key points throughout the document. Our responses below highlight a number of specific changes made in response to the minor comments provided. Reviewer comments are in bold, with our response to each below. New content from the paper is in quotes. As requested, these revisions are also highlighted in the revised manuscript.

We thank the reviewers for leading us to improve clarity of this piece, and trust that this revised draft presents a more intuitive and clear explanation of the data and findings. Thank you again, and we look forward to any further comments you might have.

Sincerely,

The Authors

Journal Requirements:

**1. Please review your reference list to ensure that it is complete and correct. If you have cited papers that have been retracted, please include the rationale for doing so in the manuscript text, or remove these references and replace them with relevant current references. Any changes to the reference list should be mentioned in the rebuttal letter that accompanies your revised manuscript. If you need to cite a retracted article, indicate the article’s retracted status in the References list and also include a citation and full reference for the retraction notice.**

Thank you for this reminder. We have reviewed the references and corrected several errors:

1. Reference 2: corrected page numbers from 1-9 to 24.
2. Reference 3: corrected order of authors so that Yamey is third author and Mao is fourth.
3. Reference 3: corrected title capitalization, making several words lowercase
4. Reference 3: added missing words from title: “Version 2.0”.
5. Reference 3: corrected issue number and page number; page number is now placed where it should be
6. Reference 9: replaced inaccurate issue number and page number with article number
7. Reference 10: replaced inaccurate issue number and page number with article number
8. Reference 14: edited to include full name of journal: “China: An International Journal”
9. Reference 15: edited to include full name of journal: “China: An International Journal”
10. Reference 16: added semicolon between publishing date and issue number
11. Reference 17: replaced inaccurate issue number and page number with article number
12. Reference 18: removed repeated information, reorganized to maintain consistency with other references
13. Reference 22: replaced inaccurate issue number and page number with article number
14. Reference 23: replaced inaccurate issue number and page number with article number
15. Reference 26: replaced inaccurate issue number and page number with article number

No cited papers have been retracted.

**2. Please provide separate figure files in .tif or .eps format only and remove any figures embedded in your manuscript file. Please also ensure that all files are under our size limit of 10MB.**

For more information about figure files please see our guidelines:

<https://journals.plos.org/globalpublichealth/s/figures> 
<https://journals.plos.org/globalpublichealth/s/figures#loc-file-requirement>

We have reviewed the figure files and will attach separate figure files in .tif formats that are less than 10MB.

**3. Fig 2: please (a) provide a direct link to the base layer of the map (i.e., the country or region border shape) and ensure this is also included in the figure legend; and (b) provide a link to the terms of use / license information for the base layer image or shapefile. We cannot publish proprietary or copyrighted maps (e.g. Google Maps, Mapquest) and the terms of use for your map base layer must be compatible with our CC-BY 4.0 license.**

**Note: if you created the map in a software program like R or ArcGIS, please locate and indicate the source of the basemap shapefile onto which data has been plotted.**

**If your map was obtained from a copyrighted source please amend the figure so that the base map used is from an openly available source. Alternatively, please provide explicit written permission from the copyright holder granting you the right to publish the material under our CC-BY 4.0 license.**

Thank you for allowing us to clarify. The lead author created the maps in ArcGIS 10.7.1 using basemaps from geoBoundaries. We have updated figure 2 to include this as a note as well as included the appropriate references in the narrative as follows:

Fig 2 was created in ArcGIS 10.7.1 using basemaps from geoBoundaries. (22,23)

**Reviewer #1:**

**Intro: Consider adding additional context, specifically- how does this $ amount of aid compare to other countries’ health aid? How does China’s health aid compare to other infrastructure projects? (e.g. dams, tunnels, etc)**

Thank you for this suggestion. We thoughtfully considered it with the following data points:

Health projects account for 13% of China's aid portfolio.

<https://gh.bmj.com/content/7/12/e010408.long>

"China was the fifth largest health aid donor in Africa from 2008 to 2017. The USA is by far the largest bilateral health donor in African countries, followed by the UK. However, China had very similar levels of health aid to Germany, the Netherlands and Japan."

<https://gh.bmj.com/content/7/12/e010408.long>

Given the variation in both timing and methodology we decided that adding the points would create more confusion and less clarity for the reader. Therefore, we focused our efforts on the suggestions to make the sourcing of our data clearer and more concise.

**Line 93: “China…. does not typically align with countries’ national and subnational health priorities” – consider giving an example/evidence either here or in the discussion.**

We resolved this sentence to exclude the second half since it was not relevant to the point being made.

**Line 163-164: DAC codes – what does DAC stand for? Define ODA and OOF (need to include full name before abbreviation)**

Thank you for letting us know we did not clearly define some of the acronyms. We have included the full times as follows:

“The data are categorized as Official Development Assistance (ODA)-like, Other Official Flows (OOF)-like, or Vague (official Finance) based on Organisation for Economic Co-operation and Development Assistance Committee (OECD-DAC) guidelines for ODA and OOF.”

**Line 143-156: Consider including single sentence about where GCDF pulls data from (there is a good explanation in the discussion section, but it leaves readers wondering for the entire paper where this data is sourced from). Also consider sentence on what type of organization AidData is (non-profit? Government-affiliated? Based in a single country?)**

We have updated the narrative to make it clearer and more precise. We included this specific language:

“…this is done by organizing extensive official source catalogs for each country, backed by media sources, from where trained staff create project records which later undergo mutli-stage data quality reviews.”

“AidData is a non-profit research institution based at William & Mary, a research university based in Virginia, United States .”

**Line 163-164: Exclusion of OOF-like and vague – how does this change the results? (consider addressing in limitations section)**

This is an important point. We have clarified the exclusion criteria with the following sentence**:**

“Only 26 projects in GCDF, or 2.4% of all health projects in Africa, fall into the OOF and vague categories, nearly all of which are export buyer’s credits for medical equipment purchase.”

**Line 167: What was the “detailed protocol” for reclassifying projects? It might be helpful to show an example or a flowchart of the protocol**

We have updated the bibliography to include a citation of source of the AidData protocol so the reader can easily reference the detailed protocol.

“Organisation for Economic Co-operation and Development DAC Working Party on Development Finance Statistics. Converged Statistical Reporting Directives for the Creditor Reporting System (CRS) and the Annual DAC Questionnaire Annexes – modules A, B, and C. 2021 20 April”

In addition, we clarified the paragraph as follows:

“Based on the GCDF 2.0 and OECD-DAC guidelines, we built on the GCDF 2.0 data and designed a detailed protocol for reclassifying all 1,026 health projects in Africa from their broad 3-digit OECD-DAC sector codes to their more specific 5-digit CRS codes. We did this by following OECD’s sector classification guidelines, which provide guidelines and detailed nesting information of 5-digit codes within each of the 24 sector categories represented at the 3-digit code level. For example, whereas all health sector (code: 120) projects are 3-digit tagged, activities most closely aligned with “tuberculosis control” (code: 12263) and “basic health infrastructure” (code: 12230) are allocated their respective 5-digit codes. We followed a double-blind review process for coding health projects into 5-digit codes for sub-sectors, i.e., each project was reviewed by two independent coders to ensure internal consistency. In cases with discrepancies, plus an additional 10% of the sample picked randomly, senior coders undertook additional quality assurance processes by reviewing coder determinations for consistency and accuracy. To compare China to more traditional global health donors, project-level data from CRS (purpose codes 12110, 12181, 12182, 12191, 12220, 12230, 12240, 12250, 12261, 12262, 12263, and 12281) were also obtained for the years 2000–2017 from the OECD web portal (accessed April 2022). “

**Line 205-208: How were “resource-rich” countries defubed? By GDP? Was there a standardized metric? Recommend including their definition/method of determination of “resource-rich”**

We have changed this sentence to be more clear and precise. It now reads:

“…we find that certain countries rich in natural resources like Angola and DRC are getting the lion's share of commitments over this long commitment period.”

**Line 219-220: Consider further defining “basic health care” and “medical services” projects? Vaccines, medicines, drugs and vertical programs like Malaria much more intuitive. How do basic health care and medical services differ from each other?**

This is a confusing part of the OECD guidelines and one I wish were more transparent. To increase the transparency in our narrative we included the following sentence:

“In our analysis we included the categories of “basic care” and “medical services” which are similar, but not identical. Basic care refers to directly delivering health services and training local staff; whereas “medical services” refers to primarily medical equipment and staffing. Projects involving activities like “nursing home” do not fit into basic health since it is specialized and therefore were coded as medical services”

**Line 226: How many projects (what percent) missing financial data? Why do some projects have commitments valued and some not? Are there any trends? How could this skew/bias data? (Consider further discussion limitations)**

661 projects, out of 1026 projects, which are 64.4% of projects that are currently missing transaction amounts. The reason is simple, those data are not publicly available and AidData was not able to retrieve and record that information in the GCDF2.0 dataset. Mostly we are missing primary code 12201 projects, which are medical team-related projects. Due to the nature of those projects, unless official sources clearly mentioned the transaction amount, it’s very hard to find the actual commitment value or make estimations. Since their operational cost varies in different countries and different years for different purposes. This implies that readers should consider the reported financial values of China's health interventions in Africa as underestimates. There is no way to improve the data coverage unless one day in future China would release all audited government budgets related to those project

**Line 240-241: Do not agree that increasing scale and reach of malaria projects would “indicate the feasibility of Chinese malaria control projects in Africa.” Please clarify/re-word.**

We agree this assumption is too strong. We have updated the narrative to the following:

**“**Their interest in malaria also expanded in scale and depth between 2006-2009, indicating a sustained commitment to Chinese malaria control projects in Africa.” **Line 251-253: Sentence could be improved for clarity. Furthermore, please clarify who DAC members are.**

Thank you for the opportunity to clarify. The sentence now reads:

“In contrast to Fig 3, which focuses on China, Fig 4 shifts focus to examine the allocation of health aid among the 30 DAC members across all Africa countries over the same 18-month period. The primary purpose of Fig 4 is to explore the allocation of health aid among the DAC members.” **Line 257-259: Please rewrite sentence for clarity**

Thank you again for the opportunity to clarify. The sentence now reads:

**“**Both China and the OECD health aid projects have maintained a steady trend in their total allocations since 2009 when total allocations rose from under $2 billion to approximately $2.5 billion per year.” **Line 331: Please write out SDG, BRI, FOCAC**

Thank you for highlighting these acronyms that we did not clearly define. The narrative now reads as follows:

**“**In addition, the paper highlights China’s intentions to prioritize boosting public health systems in Africa and to uphold its commitments to achieve targets: Sustainable Development Goals (SDGs), China’s Belt and Road Initiative (BRI), and the Forum on China-Africa Cooperation (FOCAC). “

**Line 334: What kinds of “official sources”?**

This line has been updated to the following:

“These include original unredacted loan agreements, government reports from Chinese implementation agencies or recipient country departments, or government press releases.”

In addition, we included a citation to the methodology: <https://scholar.google.com/citations?view_op=view_citation&hl=en&user=RLiLArUAAAAJ&citation_for_view=RLiLArUAAAAJ:AXPGKjj_ei8C>

**Line 351-352: Interesting comment about differences between aid sectors. Consider further supporting this statement with an example.**

**We have updated the narrative to include two specific examples from their research. The narrative now reads:**

**“…**yet Guillon & Mathonnat observe significant differences between aid sectors, including that social aid is more responsive to the economic needs of recipient countries and is also influenced by foreign policy considerations. (30) Research could seek to build upon their findings.”

**Figures –make sure Y axis labeled on all figures with appropriate title + units, also make sure all Figure titles are as clear/descriptive as possible (ex. Fig 4 title could be more specific about including 30 DAC countries)**
**- Figure 2 (Does Right Map reflect “Number of Chinese Aid Projects” or is it “Number of Chinese Health Aid Projects” – if it is the former it might help to further specify that it is “All Project Types” or if it is the latter, recommend including the word Health to clarify)**
**- Figure 3 – make sure y axis is completely labeled (% of what). Also, what is “Basic Care”?**
**- Figure 4 – label Y axis, clarify in text how are basic healthcare and medical services different? Why is TB separate from other infectious disease?**

Thank you for these suggestions. We have updated the figures to better clarify the axis as well as titles. The figures are now in color based on a suggestion from reviewer #2. We also clarified the difference between basic healthcare and medical services in the narrative.

**Reviewer #2: The article demonstrated evidence-based review and analysis. It actually met the publication criteria of PLOS GLOBAL PUBLIC HEALTH.**

**My observation however, is that, the authors would have presented the figures in color ink to bring out the significance of the points and inferences. Is it possible to achieve this before final publication? I wish to strongly suggest this be given a thought.**

Thank you for this suggestion. We have updated the figures to be in color.

**Others:**
**Results: Line 1 200-2017. Please, replace it with 2000-2017.**

Thank you for highlighting this mistake.

**The same is applicable to number 63. Please, edit accordingly.**

Thank you for highlighting this mistake. We have fixed line 63 to read as follows:

**“**China's priorities have shifted over time (2000-2017).”
